# On the Farcino-Glandulous Disease in Man

**Published:** 1848-06

**Authors:** 


Art. X.—*On the Farcino-Glandulous Disease in Man.* By Dr. A. Marchant, of Charenton, near Paris, France. Translated for this Journal by E. Brooks, Jr. of Boston.

Veterinary Physicians have given the names *Glanders* and *Farcy* to two diseases which appear to have the same origin, but which differ from each other in their external forms and nosological characters. They are spontaneously developed in solipedes under the influence of more or less known causes and can be thence transmitted to man either by contagion or infection.

It was thought until these latter years that solipides alone could be attacked with glanders and farcy—but since the labors of Mr. Elliotson in England, (1) and of M. Rayer in France, (2) all accomplished physicians and veterinarians are satisfied that man can contract these diseases either by the contact of or by cohabitation with glandered and farcied animals. Examples of the disease have multiplied in proportion as it has become better known and new observations of it are daily made public by the medical journals. The contagion exercises itself much more frequently from the horse to other solipedes than from the horse to man—but it is not the less real that it be rare, and this truth has been gained to science by so large a number of facts that it is no longer possible to establish the least doubt respecting it.

What name then should be given to the human glanders and farcy? Shall we adopt the denomination Equinia (4) proposed by Mr. Elliotson, or that of glanders, affection morveuse, (5) adopted by MM. Bérard and Denonvilliers (3). The former indicates the origin and comprehends the two principal forms of the disease but it introduces without an acknowledged necessity, a new term into a science, the glossology of which is already very extensive. The term glanders adopted by MM. Bérard and Denonvillieres is incomplete for it expresses but one form of the disease; it would be more logical to call the two diseases united by the name of the farcino-glandulous disease. Although this denomination be a somewhat long one, it has the great advantage of designating the whole disease without giving rise to the least equivocation.

I will add that the farcino-glandulous disease is specifiic and contagious, and is produced by the introduction of a peculiar morbid principle—a true animal poison—into the economy.

Specific and contagious diseases present two principal varieties. Those of the first are distinguished by a nearly constant regularity in their march, which enables the physician to foresee and to announce it in its details with an almost mathematical precision—apart from some exceptional cases when these diseases present what is called an abnormal form—variola, rubiola, scarlatina, &c., belong to this category.

The diseases of the second variety, though produced by a like virulent cause, have less of conformity and regularity in their manifestation—thus syphilis, which may be placed in this class, presents (6) primitive, secondary, local, and general, or constitutional affections, which at the first glance appear to be so many particular morbid states, and which are, notwithstanding, only several expressions of the venereal disease.

The syphilitic virus when introduced into the human body produces lesions of the mucous membranes (blenorrhagia, chancres)* alterations of the osseous system (exostosis, osteocopus) inflammation of the lymphatic ganglia, &c., (bubos). The cause of all these morbid expressions is evident, and no judicious physician hesitates to pronounce upon the existence of the venereal disease so soon as one or several of these symptoms shall have appeared.

The farcino-glandulous poison, always identical in its origin and nature, may in like manner produce either glanders or farcy, or what is more common, the two affections united.

This comparison between syphilis and the farcino-glandulous disease had already been made by veterinary physicians, and from the fact that these two diseases present some points of resemblance, they concluded that they were identical, but veterinarians soon perceived their error and are now generally satisfied of the individuality of the disease.

---

* Lesions of the external tegument (Syphilides, Excrescences.)

MM. Bérard and Denonvilliers appear to partake of the opinion expressed above, when they say (7) "syphilis has numerous relations with glanders, since like it, it manifests itself by pains, tumors, ulcerations and a variety of alterations which are situated in the nasal cavities, the throat, the respiratory passages, the osseous and cutaneous systems."

These resemblances are much more evident when the two diseases are compared intimately together; it is then seen that they offer the same irregularity in the march of their symptoms, and this manner of considering the *f. g.* disease evidently leads to the formation of the following proposition: That farcy and glanders are but manifestations of the *f. g.* poison in the economy—there are two complex symptoms of poisoning, but not two distinct diseases.

In this memoir I shall only occupy myself with the *f. g.* disease in man—such as it has been observed until now by physicians. Science already possesses a large number of observations which when analyzed with care, tend to prove that the forms of this disease as observed by veterinary physicians are not absolutely the same in man. I therefore propose for the *f. g.* disease in man the following nosological classification.

- Farcino-glandulous disease.
  - Acute glanders.
  - Farcy.
    - Simple.
    - Grave.
      - 1st. Without chronic alteration of the nasal cavities, and most commonly ending in acute glanders.
      - 2dly. With chronic alteration of the nasal cavities, (chronic glanders.)

This classification appears to me to be more natural and true than that which physicians have borrowed from Veterinarians. Indeed acute glanders, which has till now been rapidly mortal in man, cannot, consequently, be compared to chronic glanders which rather resembles farcy in its duration and symptoms—this will be amply proved in the course of my memoir.

It is under the form of acute glanders that the greatest violence of farcino-glandulous poisoning manifests itself. Whether preceded by farcy or not, it has till now always proved fatal to the individual affected with it. Farcy is slower in its development, and goes through its periods with more or less rapidity, according to the resistance with which it meets in the organization of man; it destroys and ruins, little by little, and the remissions observed in this disease seem to prepare the organization for the apparition of acute glanders which, generally speaking, ends at once the suffering and lives of our unfortunate farcy patients.

The division of farcy into acute and chronic, should be absolutely discarded, because the signs which serve to establish it are not sufficiently certain, and lead to no important practical results.

Indeed, is it upon the duration of the disease that this dichotomy can be founded? how difficult would it be to determine at what period the acute state ended, and when the chronic state began! Is it upon the march of the symptoms? this again is impossible, since abscesses exist in chronic farcy which take the phlegmonous form. It is wiser then to study and understand farcy in the light presented above, since the divisions heretofore established are sometimes confounded with each other, and are the source of no therapeutical indications.

Farcy is sometimes susceptible of cure; science it is true possesses some rare examples of this. It therefore becomes necessary to admit a variety of farcy uncomplicated with acute glanders. I have called it *simple* farcy, which indicates the disease in a precise manner. Unfortunately the signs which serve to distinguish it from *grave* farcy are not prominent enough to be pronounced upon. But it is to be hoped when once the attention of physicians shall be directed to this point, that more certain means of distinguishing between these two varieties of farcy will be discovered.

The physicians who first wrote upon farcy and glanders borrowed of Veterinarians the theories which they had formed upon these diseases, and they labored to include all cases of the f. g. disease in the divisions admitted by veterinary medicine; but the observations which have been collected of chronic glanders in man differ wholly from the same disease as it is witnessed in horses, in whom it often makes its appearance without, so to speak, altering their general health; they preserve their *embonpoint* and render good service to their owners, who continue to work them as if they were free from all complaint. Now, it is wholly otherwise in man, in whom chronic glanders is never simple and exempt from farcy. In M. A. Tardieu's (8) observations, given as an instance of chronic glanders without farcy, there was an abscess on a level with the fourth metatarsal on the left side, proving in an evident manner the existence of farcy as well, since this disease is characterized in man by more or less numerous abscesses.

---

NOTES.—(1) Elliotson on the glanders in the human subject; Medico-Chirurgical Transactions, vol. xvi., pp. 1, 171.

(2) Rayer, De la Morve et du Farcin chez l'homme. Paris, 1837.

(3) Bèrard and Denonvilliers; Compendium de Chir. Prat., p. 509.

(4) Mr. Elliotson proposes the generic name of *Equinia*, which then divides into species—Eq nasalis for glanders, Eq. apostimatos for farcy.

(5) I should here remark, for the sake of lucidity, that the French word *Morva* designates in reality the same disease as does the English word glanders; yet that their origins are wholly different. Morva literally signifies *snot*, or in veterinary medicine, that discharge from the nostrils which is peculiar to the disease called glanders. Whereas glanders is descriptive of that enlargement of certain lymphatic *glands*, which is but another symptom of the disease. The French have christened the disease from one, and the English from another, of its prominent characteristics. The English word farcy is a modification of the French word *farcin*, (from *farcire* to stuff), I have therefore rendered the expression *farcino-morvous*, farcino-glandulous.—E. B., jr.

(6) Dict. de Med. et Chir. Prat., tome xv., p. 176.

(7) Compendium de Chirurgic Pratique, tome i,, p. 541.

(8) A. Tardieu, de la Morve et du Farcin Chronique, &c., p. 121.

